# Floristic Analysis of Vascular Plants in the Ziwuling Mountains, Shaanxi Province

**DOI:** 10.3390/plants14071006

**Published:** 2025-03-23

**Authors:** Shuyue Ma, Fangfang Qiang, Guangquan Liu, Changhai Liu, Chongyan Bai, Ning Ai

**Affiliations:** 1 Key Laboratory of Applied Ecology on the Loess Plateau of Shaanxi Higher Education Institutions, College of Life Sciences, Yan’an University, Yan’an 716000, China; mashuyue0000@yau.edu.cn (S.M.); ffqiang@yau.edu.cn (F.Q.); yadxlch@yau.edu.cn (C.L.);; 2 China Institute of Water Resources and Hydropower Research, Beijing 100038, China; gqliu@iwhr.com

**Keywords:** vascular plant, Ziwuling Mountains, flora, geographical components, plant taxonomy

## Abstract

A study was conducted on the vascular floras of the Ziwuling Mountains in Shaanxi Province to establish a foundational database, providing data support for the conservation and utilization of the Ziwuling Mountains’ plant diversity resources. Based on field surveys and literature references, the composition and geographical elements of the vascular floras in the study area were analyzed. The species richness and floristic similarity coefficients of the study area were compared with other floras on the Ordos Platform. The results were as follows: (1) The vascular floras of the Ziwuling Mountains in Shaanxi Province comprised 120 families, 498 genera, and 965 species, with superrosids and superasterids being significantly dominant. There were 15 dominant families, primarily composed of oligotypic and monotypic genera. In terms of life forms, perennial forbs were the most abundant. (2) At the family level, tropical elements slightly outweighed temperate elements; at the genus level, temperate elements dominated. (3) The study area was rich in rare and endangered species. (4) Compared with other floras on the Ordos Platform, the study area exhibited higher species richness and the greatest similarity with the Liupan Mountain floras. The biodiversity of the Ziwuling Mountains in Shaanxi Province was relatively high, with diverse geographical elements and abundant rare and endangered species resources.

## 1. Introduction

China is one of the countries with the highest plant diversity richness in the world [1,2,3]. In recent years, the conservation of plant diversity has become one of the most widely discussed global issues [4,5,6,7]. Protecting plant diversity is the cornerstone of maintaining ecosystem stability, safeguarding essential resources for human survival and addressing global challenges such as climate change. Flora refers to the totality of plant species in a specific region, period, taxon, or vegetation type, encompassing the sum of plant families, genera, and species [8,9]. It not only provides insights into the natural historical development and environmental changes of plants in a region but also offers foundational data for the conservation of plant diversity and the sustainable utilization of plant resources [10]. The Ziwuling Mountains in Shaanxi Province are located in the heart of the Loess Plateau. Although numerous studies have been conducted on the floras of the Loess Plateau, research on the vascular floras of the Ziwuling Mountains in Shaanxi Province remains limited [11,12]. Notably, the “Liupan-Ziwuling Mountain region” was designated a key conservation area in China’s Biodiversity Conservation Priority Areas plan promulgated in 2015. However, the baseline data on plant diversity in the Ziwuling area within this region remain incomplete, which significantly hinders the targeted implementation of conservation measures. Therefore, it is essential to conduct a comprehensive study on the vascular floras of the Ziwuling Mountains in Shaanxi Province. This research investigates the vascular plant resources of the Ziwuling Mountains and systematically analyzes its floras, with the aim of establishing a baseline database for the vascular plant diversity of the Ziwuling Mountains in Shaanxi Province. Additionally, it provides data support for the conservation and utilization of plant diversity in the northern Shaanxi Loess Plateau, thereby promoting ecological improvement and high-quality development in the region.

## 2. Results and Analysis

### 2.1. Composition of Vascular Plant Floras

**Figure 1:**
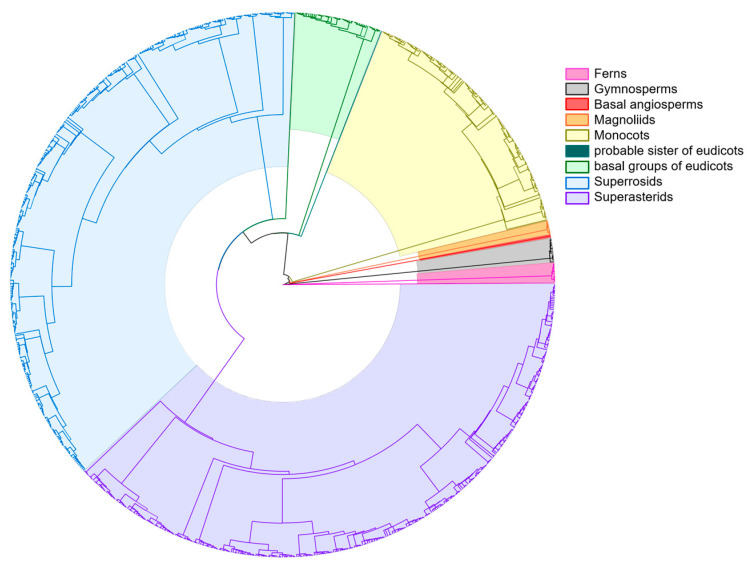
The species composition of vascular plants in the Ziwuling Mountains of Shaanxi Province.

According to the survey statistics and data processing (Figure 1), the vascular plants in the Ziwuling region of Shaanxi Province comprised 965 species (498 genera and 120 families), including ferns (5 families, 8 genera, and 12 species; 4.17%, 1.61%, and 1.24% of the total families/genera/species), gymnosperms (3 families, 7 genera, and 14 species; 2.50%, 1.41%, and 1.45%), and angiosperms classified using the APG IV [13] system, a molecular phylogenetics-based framework for monophyletic taxa. The angiosperms consisted of basal angiosperms (1 family, 1 genus, and 2 species; 0.83%, 0.20%, and 0.21%), magnoliids (4 families, 5 genera, and 8 species; 3.33%, 1.00%, and 0.83%), monocots (21 families, 90 genera, and 147 species; 17.50%, 18.07%, and 15.23%), a probable sister of eudicots (1 family, 1 genus, and 1 species; 0.83%, 0.20%, and 0.10%), basal groups of eudicots (5 families, 21 genera, and 50 species; 4.17%, 4.22%, and 5.18%), and core eudicots subdivided into superrosids (41 families, 159 genera, and 370 species; 34.17%, 31.93%, and 38.34%) and superasterids (39 families, 206 genera, and 361 species; 32.50%, 41.37%, and 37.41%).

### 2.2. Composition of Vascular Plant Families and Analysis of Dominant Families

Based on the number of species they contained, the families were categorized into the following five levels: monotypic family (containing 1 species), oligotypic family (containing 2–4 species), mesotypic family (containing 5–9 species), relatively large family (containing 10–19 species), and big family (containing 20 or more species) (Table 1). Among these, big families included 7 families, 191 genera, and 396 species, such as Asteraceae, Rosaceae, Fabaceae, and Poaceae, accounting for 5.83%, 38.35%, and 41.04% of the total families, genera, and species of vascular plants in the study area, respectively; relatively large families included 19 families, 132 genera, and 256 species, such as Amaranthaceae, Cyperaceae, Asparagaceae, and Brassicaceae, accounting for 15.83%, 26.51%, and 26.53% of the total families, genera, and species of vascular plants in the study area, respectively; mesotypic families included 26 families, 78 genera, and 102 species, such as Pinaceae, Plantaginaceae, Sapindaceae, and Violaceae, accounting for 21.67%, 15.66%, and 18.03% of the total families, genera, and species of vascular plants in the study area, respectively; oligotypic families included 38 families, 67 genera, and 109 species, such as Araliaceae, Bignoniaceae, Cactaceae, and Campanulaceae, accounting for 31.67%, 13.45%, and 11.30% of the total families, genera, and species of vascular plants in the study area, respectively; and monotypic families included 30 families, 30 genera, and 30 species, such as Acoraceae, Cannaceae, Ceratophyllaceae, and Commelinaceae, accounting for 25.00%, 6.02%, and 3.11% of the total families, genera, and species of vascular plants in the study area, respectively.

In the study area, 15 dominant families were identified, comprising 255 genera and 528 species, which accounted for 51.20% of the total genera and 54.72% of the total species, respectively. These dominant families included Asteraceae (53 genera/107 species), Poaceae (38 genera/54 species), Fabaceae (31 genera/68 species), Rosaceae (22 genera/88 species), Lamiaceae (20 genera/25 species), Apiaceae (16 genera/21 species), Amaranthaceae (14 genera/18 species), Ranunculaceae (11 genera/33 species), Brassicaceae (11 genera/17 species), Cucurbitaceae (10 genera/13 species), Orchidaceae (10 genera/12 species), Polygonaceae (9 genera/16 species), Asparagaceae (8 genera/17 species), Cyperaceae (6 genera/18 species), and Caprifoliaceae (6 genera/17 species).

### 2.3. Composition of Vascular Plant Genera and Species

Based on the number of species they contained, the genera were categorized into the following four levels: monotypic genera (containing 1 species), oligotypic genera (containing 2–4 species), mesotypic genera (containing 5–9 species), and big genera (containing 10 or more species) (Table 2). Among these, big genera included 4 genera and 57 species, such as *Artemisia*, *Prunus*, *Clematis*, and *Malus*, accounting for 0.80% and 5.91% of the total genera and species of vascular plants in the study area, respectively; mesotypic genera included 37 genera and 234 species, such as *Potentilla*, *Salix*, *Viola*, and *Allium*, accounting for 7.43% and 24.25% of the total genera and species of vascular plants in the study area, respectively; oligotypic genera included 141 genera and 358 species, such as *Cynanchum*, *Lactuca*, *Leontopodium*, and *Equisetum*, accounting for 28.31% and 37.10% of the total genera and species of vascular plants in the study area, respectively; and monotypic genera included 316 genera and 316 species, such as *Acorus*, *Atriplex*, *Axyris*, and *Bassia*, accounting for 63.45% and 32.75% of the total genera and species of vascular plants in the study area, respectively.

### 2.4. Analysis of Life Forms and Growth Forms of Vascular Plants

**Figure 2:**
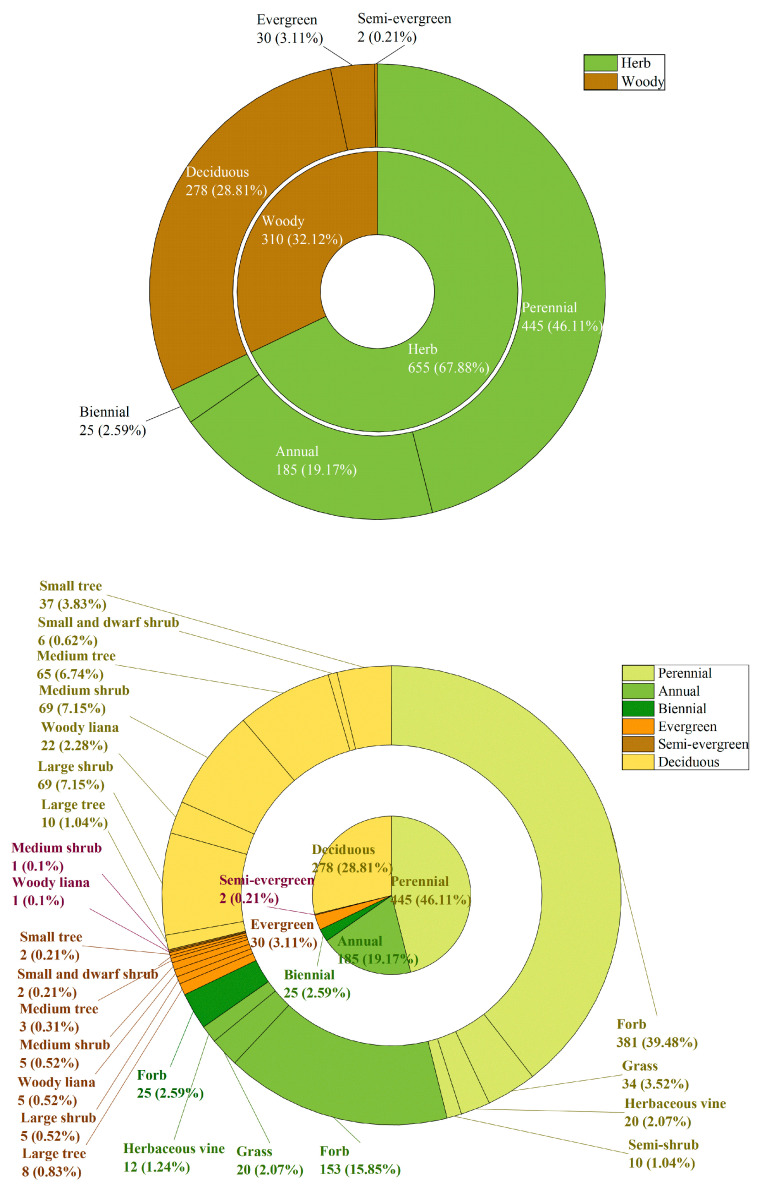
The spectrum of life forms and growth forms in the vascular floras. Note: The upper figure illustrates the life-form composition of vascular plants in Ziwuling, characterized by a predominance of herbaceous and woody plants, while the lower figure delineates the structural differentiation of growth forms within each life-form category.

The composition of life forms and growth forms of vascular plants in the study area was analyzed (Figure 2). In terms of life forms, herbs comprised 655 species (67.88% of the total species). This included 185 annual species (19.17%), 25 biennial species (2.59%), and 445 perennial species (46.11%). Woody plants comprised 310 species (32.12%). This included 30 evergreen species (3.11%), 2 semi-evergreen species (0.21%), and 278 deciduous species (28.81%).

When further subdivided by growth forms, annual forbs included 153 species (15.85% of the total species), annual grasses included 20 species (2.07%), annual herbaceous vines included 12 species (1.24%), biennial forbs included 25 species (2.59%), perennial forbs included 381 species (39.48%), perennial grasses included 34 species (3.52%), perennial herbaceous vines included 20 species (2.07%), and perennial semi-shrubs included 10 species (1.04%).

Among the woody plants, evergreen large trees included 8 species (0.83% of the total species), evergreen large shrubs included 5 species (0.52%), evergreen woody lianas included 5 species (0.52%), evergreen medium shrubs included 5 species (0.52%), evergreen medium trees included 3 species (0.31%), evergreen small and dwarf shrubs included 2 species (0.21%), and evergreen small trees included 2 species (0.21%). Semi-evergreen woody lianas included 1 species (0.1%), semi-evergreen medium shrubs included 1 species (0.1%), deciduous large trees included 10 species (1.04%), deciduous large shrubs included 69 species (7.15%), deciduous woody lianas included 22 species (2.28%), deciduous medium shrubs included 69 species (7.15%), deciduous medium trees included 65 species (6.74%), deciduous small and dwarf shrubs included 6 species (0.62%), and deciduous small trees included 37 species (3.83%).

### 2.5. Geographical Elements of Seed Plants

#### 2.5.1. Distribution Types of Families

According to Wu Zhengyi’s classification of distribution types, the seed-plant families in the Ziwuling region were divided into 15 distribution types (Table 3). Within the study area, Cosmopolitan families included 44 families, accounting for 38.60% of the total seed-plant families, such as Alismataceae, Amaranthaceae, Apiaceae, and Asteraceae.

Excluding Cosmopolitan families, tropical distribution types (Types 2–7) comprised 37 families, accounting for 52.86%. Among these, Pantropic included 27 families (38.57%), such as Anacardiaceae, Apocynaceae, Araceae, and Aristolochiaceae; Trop. Asia, Africa & C. to S. Amer. disjuncted included Iridaceae (1 family; 1.43%); Pantropic Trop. especially S. Hemisphere included 3 families (4.29%), such as Amaryllidaceae, Loranthaceae, and Phytolaccaceae; Trop. Asia & Trop. Amer. disjuncted included 3 families (4.29%), such as Araliaceae, Cactaceae, and Nyctaginaceae; Old World Tropics included 2 families (2.86%), such as Asparagaceae and Pedaliaceae; and S. Africa included Ericaceae (1 family; 1.43%).

Temperate distribution types (Types 8–12) comprised 32 families, accounting for 45.71%. Among these, North. Temperate included 7 families (10.00%), such as Cannabaceae, Caprifoliaceae, Hypericaceae, and Liliaceae; N. Temper. & S. Temp. disjuncted included 13 families (18.57%), such as Betulaceae, Cornaceae, Cupressaceae, and Elaeagnaceae; Eurasia & Temp. S. Amer. disjuncted included Berberidaceae (1 family; 1.43%); E. Asia & N. Amer. disjuncted included 8 families (11.43%), such as Acoraceae, Magnoliaceae, Nelumbonaceae, and Penthoraceae; Old World Temperate included Tamaricaceae (1 family; 1.43%); Mediterranean to C. & S. Africa, Australasia disjuncted included Asphodelaceae (1 family; 1.43%); and Mediterranean to Temp. -Temp. Asia, Australasia & S. Amer. disjuncted included Nitrariaceae (1 family; 1.43%). Additionally, there was 1 family Endemic to China, Ginkgoaceae, accounting for 1.43%.

#### 2.5.2. Distribution Types of Genera

According to Wu Zhengyi’s classification of distribution types for seed-plant genera, the seed-plant genera in the Ziwuling region were divided into 28 distribution types (Table 3). Within the study area, Cosmopolitan genera included 63 genera, accounting for 13.32% of the total seed-plant genera, such as Agrostis, Alkekengi, Amaranthus, and Apium.

Excluding Cosmopolitan genera, tropical distribution types (Types 2–7) comprised 109 genera, accounting for 26.59%. Among these, Pantropic included 47 genera (11.46%), such as Abutilon, Aristida, Aristolochia, and Boehmeria; Trop. Asia, Australasia & C. to S. Amer. disjuncted included Nicotiana (1 genus; 0.24%); Trop. Asia & Trop. Amer. disjuncted included 19 genera (4.63%), such as Agave, Arachis, Canna, and Capsicum; Old World Tropics included 10 genera (2.44%), such as Albizia, Asparagus, Dracaena, and Flueggea; Trop. Asia, Africa & Australasia disjuncted included Thesium (1 genus; 0.24%); Tropical Asia & Trop. Australasia included 8 genera (1.95%), such as Ailanthus, Hydrilla, Lagerstroemia, and Leptopus; Trop. Asia & Trop. Africa included 16 genera (3.90%), such as Aloe, Arthraxon, Citrullus, and Clivia; and Trop. Asia included 7 genera (1.71%), such as Benincasa, Broussonetia, Epipremnum, and Goodyera.

Temperate distribution types (Types 8–12) comprised 256 genera, accounting for 62.44%. Among these, North. Temperate included 113 genera (27.56%), such as Acer, Achillea, Aconitum, and Actaea; Arctic-Alpine included 2 genera (0.49%), such as Braya and Rhodiola; N. Temper. & S. Temp. disjuncted included 23 genera (5.61%), such as Adenocaulon, Alisma, Arenaria, and Bromus; Eurasia & Temp. S. Amer. disjuncted included 3 genera (0.73%), such as Alopecurus, Leontopodium, and Leymus; E. Asia & N. Amer. disjuncted included 28 genera (6.83%), such as Acorus, Agastache, Ampelopsis, and Amphicarpaea; Old World Temperate included 45 genera (10.98%), such as Achnatherum, Adenophora, Ajuga, and Alcea; Mediterranean. W. Asia (or C. Asia) & E. Asia disjuncted included 8 genera (1.95%), such as Rhaponticum, Takhtajaniantha, Forsythia, and Hyoscyamus; Mediterranean & Himalayan disjuncted included 2 genera (0.49%), such as Cedrus and Pentanema; Eurasia & S. Africa disjuncted included 4 genera (0.98%), such as Cnidium, Lactuca, Medicago, and Scabiosa; Temp. Asia included 12 genera (2.93%), such as Axyris, Campylotropis, Caragana, and Diarthron; Mediterranean, W. Asia to C. Asia included 11 genera (2.68%), such as Anchusa, Bassia, Calendula, and Chorispora; Mediterranean to C. Asia & Mexico to S. USA. disjuncted included 2 genera (0.49%), such as Gypsophila and Peganum; and Mediterranean to Temp. -Temp. Asia, Australasia & S. Amer. disjuncted included 3 genera (0.73%), such as Erodium, Glycyrrhiza, and Pistacia.

Additionally, C. Asia included 5 genera (1.22%), such as Cannabis, Leptopyrum, Orychophragmus, and Sphallerocarpus; C. Asia to Himalayas & SW. China included Incarvillea (1 genus; 0.24%); E. Asia included 12 genera (2.93%), such as Bothriospermum, Caryopteris, Codonopsis, and Deutzia; Sino-Japan (SJ) included 6 genera (1.46%), such as Atractylodes, Callistephus, Crepidiastrum, and Hemiptelea; Sino-Himalaya (SH) included 12 genera (2.93%), such as Dicranostigma, Hemipilia, Platycladus, and Prinsepia; and Endemic to China included 9 genera (2.20%), such as Anemarrhena, Bolbostemma, Ginkgo, and Ostryopsis.

### 2.6. Rare and Endangered Species

The floras of the study area included the following rare and endangered species (Table 4). According to the List of National Key Protected Wild Plants in China (2021), there were 2 species classified as National Grade I Protected Wild Plants in the study area, *Ginkgo biloba* and *Paeonia rockii*. Additionally, there were 9 species classified as National Grade II Protected Wild Plants. These were *Nelumbo nucifera*, *Paeonia jishanensis*, *Prunus kansuensis*, *Prunus armeniaca*, *Rosa rugosa*, *Glycine max subsp. soja*, *Glycyrrhiza uralensis*, *Oryza sativa*, and *Cypripedium franchetii*.

According to the China Biodiversity Red List—Higher Plants Volume (2020), the study area harbored the following 3 species classified as Endangered (EN), facing a high risk of extinction: *Pinus bungeana*, *Rosa rugosa*, and *Ginkgo biloba*. There were 11 species classified as Vulnerable (VU), facing a moderate risk of extinction. These were *Larix gmelinii* var. *principis-rupprechtii*, *Paeonia jishanensis*, *Caragana purdomii*, *Cypripedium franchetii*, *Cedrus deodara*, *Asarum heterotropoides*, *Juglans regia*, *Anemarrhena asphodeloides*, *Magnolia liliiflora*, *Epimedium brevicornu*, and *Pinus sylvestris var. mongholica*. Furthermore, there were 12 species classified as Near Threatened (NT), which were potentially at risk and could become threatened in the future. These were *Malus honanensis*, *Acer davidii*, *Polygonatum megaphyllum*, *Liparis fargesii*, *Juniperus rigida*, *Androsace erecta*, *Prunus armeniaca* var. *armeniaca*, *Glycyrrhiza uralensis*, *Euphorbia fischeriana*, *Carex pseudocyperus*, *Polygonatum cirrhifolium*, and *Herminium monorchis*. Additionally, there were 138 species Endemic to China.

### 2.7. Floras of the Ziwuling Mountains in Comparison to Floras of Adjacent Areas

#### 2.7.1. Comparison of Species Richness

The species richness of the study area was compared with that of the Nanniwan Wetland Park and four mountain ranges located at the boundaries of the Ordos Platform using the species density (Table 5). The results were as follows: Wula Mountain had a species density of 57.18 species/km^2^; Helan Mountain National Nature Reserve in Ningxia had a species density of 92.12 species/km^2^; Liupan Mountain had a species density of 121.20 species/km^2^; Yunqiu Mountain had a species density of 91.45 species/km^2^; Nanniwan Wetland Park had a species density of 198.67 species/km^2^; and Ziwuling had a species density of 101.37 species/km^2^.

#### 2.7.2. Comparison of Floristic Similarity

**Figure 3:**
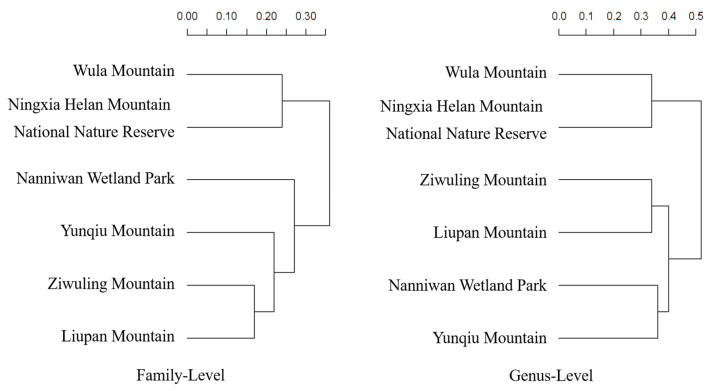
Family-level and genus-level similarity clustering of adjacent floras.

Based on the comparison of similarity coefficients (Table 6 and Figure 3), the study area showed the highest similarity with Liupan Mountain at both family and genus levels, with coefficients of 83.33% and 65.74%, respectively. The second-highest similarity was observed for Yunqiu Mountain, with coefficients of 78.40% at the family level and 63.29% at the genus level. The third-highest similarity was with Nanniwan Wetland Park, with coefficients of 73.21% at the family level and 61.68% at the genus level. The fourth-highest similarity was with the Helan Mountain National Nature Reserve in Ningxia, with coefficients of 65.45% at the family level and 49.50% at the genus level. The lowest similarity was with Wula Mountain, with coefficients of 64.15% at the family level and 48.00% at the genus level.

## 3. Discussion

The vascular floras of Shaanxi Ziwuling comprised 120 families, 498 genera, and 965 species. At the family level, superrosids and superasterids accounted for 34.17% and 32.50%, respectively; at the genus level, they accounted for 31.93% and 41.37%, respectively; and at the species level, they accounted for 38.34% and 37.41%, respectively. This indicated that superrosids and superasterids dominated the flora of the study area. In terms of family composition, large families contained a significant number of species and played an important role in the floras. Oligotypic families, medium-sized families, and relatively large families showed a gradual increase in the proportion of genera and species but a decrease in the proportion of families, suggesting that oligotypic families had a more restricted distribution and occupied more specialized ecological niches. The degree of ecological niche specialization decreased from oligotypic families to medium-sized families and then to relatively large families. Monotypic families, which were numerous, had the fewest genera and species, indicating slower evolutionary rates. The dominant families included the following 15 families: Asteraceae, Poaceae, Fabaceae, Rosaceae, Lamiaceae, Apiaceae, Amaranthaceae, Ranunculaceae, Brassicaceae, Cucurbitaceae, Orchidaceae, Polygonaceae, Asparagaceae, Cyperaceae, and Caprifoliaceae. Among these, 13 families were Cosmopolitan, while Pantropic, Old World Tropics, and North. Temperate each accounted for one family.

At the genus level, oligotypic and monotypic genera formed the main body of the vascular floras in the study area, indicating a high degree of diversification and greater diversity in the floras at the genus level [14,15,16]. Monotypic genera accounted for 63.45% of the total genera and 32.75% of the total species, while oligotypic genera accounted for 28.31% of the total genera and 37.10% of the total species. This suggests that species in oligotypic genera had broader ecological niches in recent times.

In terms of life forms and growth forms, herbs accounted for 67.88% of the total species, while woody plants accounted for 32.12%. Among the herbs, perennials dominated, and among the woody plants, deciduous species dominated. In terms of growth forms, forbs dominated among the herbs, while medium shrubs, large shrubs, and medium trees dominated among the woody plants.

An analysis of the geographical elements of seed plants in the study area revealed 15 distribution types at the family level, with Cosmopolitan families accounting for 38.60% of the total families. Excluding Cosmopolitan families, tropical distribution types accounted for 52.86%, slightly higher than the temperate distribution types. This was primarily due to the unique geographical location, habitat, and climatic conditions of the study area. Additionally, there was one family Endemic to China. At the genus level, 28 distribution types were identified, with Cosmopolitan genera accounting for only 13.32% of the total genera. Excluding Cosmopolitan genera, tropical distribution types accounted for 26.59%, while temperate distribution types accounted for 62.44%. Central Asia accounted for 1.22%, Central Asia to Himalayas & Southwest China for 0.24%, East Asia for 2.93%, Sino-Japan (SJ) for 1.46%, Sino-Himalaya (SH) for 2.93%, and Endemic to China for 2.20%. The predominance of temperate elements at the genus level aligned with the dual characteristics of the monsoon and continental climate in the study area, indicating faster speciation and broader ecological niches for temperate species in recent times.

The study revealed a rich diversity of rare and endangered species in the study area. According to the List of National Key Protected Wild Plants in China (2021), there were 2 species classified as National Grade I Protected Wild Plants and 9 species as National Grade II Protected Wild Plants. According to the China Biodiversity Red List—Higher Plants Volume (2020), there were 3 Endangered (EN) species, 11 Vulnerable (VU) species, 12 Near Threatened (NT) species, and 138 species Endemic to China.

A comparison with the seed-plant floras of adjacent regions showed that Shaanxi Ziwuling had relatively high species richness. In terms of floristic similarity, Ziwuling had the closest relationship with Liupan Mountain, suggesting a common origin [17,18,19]. The historical and modern geographical connections between the two regions are strong [20,21], likely due to the study area’s proximity to the Longshan Mountains, with the northern section of Longshan being Liupan Mountain.

## 4. Materials and Methods

### 4.1. Overview of the Study Area

The Ziwuling Mountains are located in the heart of the Loess Plateau, spanning the latitudes 33°43′ to 41°16′ N and the longitudes 100°54′ to 114°33′ E. They encompass Huanxian, Huachi, Heshui, Zhengning, and Ningxian counties in Gansu Province as well as Dingbian, Wuqi, Zhidan, Fuxian, Huangling, Yijun, Tongchuan, Yintai, Yaoxian, Chunhua, and Xunyi counties (districts) in Shaanxi Province [22]. The total area covers 23,000 square kilometers, with 12,100 square kilometers located in Shaanxi Province. The elevation ranges from 600 to 1907 m, with an average annual temperature of 9.2 °C, an annual precipitation of approximately 596.3 mm, an average annual relative humidity of 63% to 68%, and an annual sunshine duration of about 2528.4 h. The frost-free period lasts approximately 140 to 160 days [23,24]. The study area lies within the East Asian monsoon climate zone and the central Loess Plateau, resulting in a climate that exhibits both monsoon and continental characteristics. Due to the dense vegetation cover and surface runoff in the region, a localized microclimate has formed, making the overall climate of the Ziwuling area in Shaanxi more humid compared with its surroundings. The soils in the Ziwuling region of Shaanxi Province are predominantly composed of loessial soil and cinnamon soil, with minor occurrences of localized soil types such as paddy soil and saline–alkali soil. Influenced by natural topography, vegetation, climate, and human activities, the soil in the area is rich in organic matter [25], providing a solid foundation for plant diversity.

### 4.2. Plant Survey and Checklist Compilation

Based on years of field investigations and previous literature [11,12], a combination of field visits, surveys, sampling, and quadrat surveys was employed to conduct field research in the Ziwuling Mountains area of Shaanxi. The vegetation resources distributed in this region were identified, recorded, and collected. Simultaneously, referencing “*Flora of China*” [26] and the Flora of China website (http://www.iplant.cn/foc, accessed on 8 January 2025), the plant information of the area was statistically analyzed to preliminarily compile a checklist of vascular plants in the Shaanxi Ziwuling region. This U.Taxonstanveraged the Plants_WCVP database (World Checklist of Vascular Plants) [27] was integrated with the U.Taxonstand package [28] on the RStudio platform to standardize the plant nomenclature. Given that WCVP [27] serves as a core data source for Plants of the World Online (POWO) [29], species with unresolved discrepancies following automated revision via U.Taxonstand [28] were further subjected to manual verification using the official POWO platform (https://powo.science.kew.org/, accessed on 12 January 2025) [29]. For a small number of missing species in the aforementioned databases, supplementary data were incorporated based on the China Biodiversity Species Checklist (2024 Edition) from the Species 2000 China Node (http://sp2000.org.cn accessed on 14 January 2025) [30,31]. The vascular plants of the study area were classified into 9 categories using the RStudio(R 4.4.2) U.PhyloMaker [32] package and FigTree v1.4.4, including ferns and gymnosperms, and then further subdivided into angiosperms according to the APG IV [13] system, specifically basal angiosperms, magnoliids, monocots, a probable sister of eudicots, basal groups of eudicots, superrosids, and superasterids. The method for determining dominant families involved ranking the families in descending order based on the number of genera and species they contained and then identifying the families whose cumulative numbers exceeded 50%.

### 4.3. Floras, Life Forms, Growth Form Classification, and Endangered Species Determination

The distribution types of seed-plant families and genera in the study area were categorized according to the principles of the seed-plant distribution type of classification by Wu Zhengyi [33,34,35,36]. The life forms and growth forms of vascular plants in the study area were classified according to Zheng Bohan [37] and visualized using Origin. Rare and endangered species were assessed based on the “List of National Key Protected Wild Plants”, 2021 edition, and the “China Biodiversity Red List—Higher Plants Volume (2020)”. A comparison of the diversity of Ziwuling floras with adjacent floras was then conducted.

The formation and development of any flora are, to some extent, related to other similar or adjacent floras [38]. Located within the Ordos Platform, the Shaanxi Ziwuling region was compared with the following five other floras: the Nanniwan Wetland Park at the center of the Ordos Platform, the Wula Mountain at the northern boundary [39], the Helan Mountain National Nature Reserve at the western boundary [40], the Liupan Mountain at the southwestern boundary [41], and the Yunqiu Mountain at the eastern boundary [42]. The comparison focused on species richness and the floristic similarity coefficient (Ss), with species richness evaluated using species density. This approach incorporated the consideration of area as an influencing factor, providing a more comprehensive comparison than simply comparing species counts. The formulas for the species density (Sd) and similarity coefficient (Ss) are as follows:

Sd = S/ln A(1)

In Formula (1), S is the number of species, and A is the area [43].

Ss = 2c/(a + b) × 100%(2)

In Formula (2), a and b represent the number of families or genera in the two regions, respectively, and c is the number of common families or genera between the two regions, excluding globally distributed types [44].

## 5. Conclusions

(1) The vascular floras of Shaanxi Ziwuling comprised 120 families, 498 genera, and 965 species, including 5 families, 8 genera, and 12 species of ferns; 3 families, 7 genera, and 14 species of gymnosperms; and 112 families, 483 genera, and 939 species of angiosperms. Overall, superrosids and superasterids dominated the flora. Monotypic and oligotypic genera formed the main body of the genus composition. Dominant families included Cosmopolitan, tropical, and temperate distribution types. The study area was rich in vascular plant resources and exhibited high plant diversity.

(2) In terms of life forms and growth forms, perennial forbs dominated among herbs, while deciduous large shrubs and medium shrubs dominated among woody plants.

(3) In terms of geographical elements, tropical characteristics slightly outweighed temperate characteristics at the family level, while temperate characteristics dominated at the genus level.

(4) The study area included 2 National Grade I Protected Wild Plants, 9 National Grade II Protected Wild Plants, 3 Endangered (EN) species, 11 Vulnerable (VU) species, 12 Near Threatened (NT) species, and 138 species Endemic to China. The region was rich in rare and endangered species.

(5) Compared with the floras of the four mountain ranges at the boundaries of the Ordos Platform and the Nanniwan Wetland Park, Shaanxi Ziwuling had higher species richness and the greatest floristic similarity with Liupan Mountain, indicating the closest phylogenetic relationship.

## Figures and Tables

**Table 1:** Composition of the number of species in different families of vascular plants in the Ziwuling Mountains of Shaanxi Province.

Classification	Family		Genus		Species	
	Number	Percentage/%	Number	Percentage/%	Number	Percentage/%
Monotypic family (1 species)	30	25.00	30	6.02	30	3.11
Oligotypic family (2–4 species)	38	31.67	67	13.45	109	11.30
Mesotypic family (5–9 species)	26	21.67	78	15.66	174	18.03
Relatively large family (10–19 species)	19	15.83	132	26.51	256	26.53
Big family (≥20 species)	7	5.83	191	38.35	396	41.04

Note: This table categorizes family-level species counts.

**Table 2:** Composition of the number of species in different genera of vascular plants in the Ziwuling Mountains of Shaanxi Province.

Classification	Genus		Species	
	Number	Percentage/%	Number	Percentage/%
Monotypic genus (1 species)	316	63.45	316	32.75
Oligotypic genus (2–4 species)	141	28.31	358	37.10
Mesotypic genus (5–9 species)	37	7.43	234	24.25
Big genus (≥10 species)	4	0.80	57	5.91

Note: This table categorizes family-level species counts.

**Table 3:** Geographical components of families and genera of seed plants in the Ziwuling Mountains of Shaanxi Province.

Areal Type	Family		Genus	
	Number	Percentage/%	Number	Percentage/%
1. Cosmopolitan	44	-	63	-
2. Pantropic	27	38.57%	47	11.46%
2-1. Trop. Asia, Australasia & C. to S. Amer. disjuncted	-	-	1	0.24%
2-2. Trop. Asia, Africa & C. to S. Amer. disjuncted	1	1.43%	-	-
2S. Pantropic Trop. especially S. Hemisphere	3	4.29%	-	-
3. Trop. Asia & Trop. Amer. disjuncted	3	4.29%	19	4.63%
4. Old World Tropics	2	2.86%	10	2.44%
4-1. Trop. Asia, Africa & Australasia disjuncted	-	-	1	0.24%
5. Tropical Asia & Trop. Australasia	-	-	8	1.95%
6. Trop. Asia Trop. Africa	-	-	16	3.90%
6d. S. Africa	1	1.43%	-	-
7. Trop. Asia	-	-	7	1.71%
8. North. Temperate	7	10.00%	113	27.56%
8-2. Arctic-Alpine	-	-	2	0.49%
8-4. N. Temper. & S. Temp. disjuncted	13	18.57%	23	5.61%
8-5. Eurasia & Temp. S. Amer. disjuncted	1	1.43%	3	0.73%
9.E. Asia & N. Amer. disjuncted	8	11.43%	28	6.83%
10. Old World Temperate	1	1.43%	45	10.98%
10-1. Mediterranean. W. Asia (or C. Asia) & E. Asia disjuncted	-	-	8	1.95%
10-2. Mediterranean & Himalayan disjuncted	-	-	2	0.49%
10-3. Eurasia & S. Africa disjuncted	-	-	4	0.98%
11. Temp. Asia	-	-	12	2.93%
12. Mediterranean, W. Asia to C. Asia	-	-	11	2.68%
12-1. Mediterranean to C. & S. Africa, Australasia disjuncted	1	1.43%	-	-
12-2. Mediterranean to C. Asia & Mexico to S. USA. disjuncted	-	-	2	0.49%
12-3. Mediterranean to Temp. -Temp. Asia, Australasia & S. Amer. disjuncted	1	1.43%	3	0.73%
T13. C. Asia	-	-	5	1.22%
13-2. C. Asia to Himalayas & SW. China	-	-	1	0.24%
14. E. Asia	-	-	12	2.93%
14SJ. Sino-Japan (SJ)	-	-	6	1.46%
14SH. Sino-Himalaya (SH)	-	-	12	2.93%
15. Endemic to China	1	1.43%	9	2.20%

Note: Excluding Cosmopolitan in percentage.

**Table 4:** List of rare and endangered species of vascular plants in the Ziwuling Mountains of Shaanxi Province.

Reference Directory	Classification	Number of Species
List of National Key Protected Wild Plants in China (2021)	First-Class	2
	Second-Class	9
China Biodiversity Red List—Higher Plants Volume (2020)	NT	12
	VU	11
	EN	3
	Endemic species to China	138

Note: First-Class protected species: highest conservation priority, with strict protection; Second-Class protected species: high conservation priority, with regulated utilization under strict supervision; NT: Near Threatened, at risk of becoming threatened in the near future; VU: Vulnerable, with a high risk of extinction in the wild; EN: Endangered, with an extremely high risk of extinction.

**Table 5:** Comparison of species density of plant floras between the Ziwuling Mountains of Shaanxi Province and other locations.

Region	Area/km^2^	Number of Species	Species Density/km^−2^
Ziwuling Mountains	12,100	953	101.37
Nanniwan Wetland Park	10.44	466	198.67
Wula Mountain	1394	414	57.18
Ningxia Helan Mountain National Nature Reserve	2062.66	703	92.12
Liupan Mountain	1400	878	121.2
Yunqiu Mountain	210	489	91.45

**Table 6:** Comparison of the flora similarity coefficients between the Ziwuling Mountains of Shaanxi Province and other locations.

Region	Family			Genus		
	Total	Same	Ss/%	Total	Same	Ss/%
Ziwuling Mountains	71	-	-	427	-	-
Nanniwan Wetland Park	41	41	73.21	228	202	61.68
Wula Mountain	35	34	64.15	198	150	48.00
Ningxia Helan Mountain National Nature Reserve	39	36	65.45	272	173	49.50
Liupan Mountain	61	55	83.33	367	261	65.74
Yunqiu Mountain	54	49	78.40	265	219	63.29

Note: Ss denotes the similarity coefficient.

## Data Availability

Data are available on request from the corresponding authors.
